# The avian‐origin H3N2 canine influenza virus that recently emerged in the United States has limited replication in swine

**DOI:** 10.1111/irv.12395

**Published:** 2016-05-18

**Authors:** Eugenio J. Abente, Tavis K. Anderson, Daniela S. Rajao, Sabrina Swenson, Phillip C. Gauger, Amy L. Vincent

**Affiliations:** ^1^ Virus and Prion Research UnitNational Animal Disease CenterAgricultural Research ServiceUSDAAmesIAUSA; ^2^ National Veterinary Services LaboratoriesAnimal and Plant Health Inspection ServiceUSDAAmesIAUSA; ^3^ Department of Veterinary Diagnostic and Production Animal MedicineCollege of Veterinary MedicineIowa State UniversityAmesIAUSA

**Keywords:** Avian influenza, canine influenza, H3N2 virus

## Abstract

Equine‐origin H3N8 has circulated in dogs in the United States since 1999. A genetically and antigenically distinct avian‐origin H3N2 canine influenza was detected in March of 2015 in Chicago, Illinois. Subsequent outbreaks were reported with over 1000 dogs in the Midwest affected followed by 23 additional states with detections within 5 months. The potential for canine‐to‐swine transmission was unknown. Experimental infection in pigs showed this virus does not replicate efficiently in swine.

## Introduction

In March of 2015 in Chicago, Illinois an H3N2 canine influenza related to the Asian H3N2 CIV caused an outbreak in dogs (http://mediarelations.cornell.edu/2015/04/12/midwest-canine-influenza-outbreak-caused-by-new-strain-of-virus/). Two established lineages of H3 canine influenza virus (CIV) have been described, of equine‐origin and avian‐origin.^1^ An H3N8 equine influenza virus (EIV) initially caused outbreaks in racing greyhounds in the United States in 2004^2^, ^3^; however, epidemiological and serological evidence indicated that an H3N8 equine virus introduction around 1999 was likely the ancestral strain of the H3N8 CIV in the United States.^4^, ^5^ While there was evidence of additional EIV transmission events into dogs in the United States,^6^ there was no evidence of sustained transmission of these other events, nor were there reports of CIV transfer back to horses or to humans.^7^ An H3N2 avian influenza virus (AIV) was detected in dogs in Asia in ~2005 and now appears to be endemic.^8^, ^9^, ^10^, ^11^ H3N2 subtype viruses are endemic in swine. Swine have also incurred multiple cross‐species incursions and occasional maintenance of new lineages. An experimental study was designed to examine whether the newly emerged H3N2 CIV from the US was capable of infecting swine to assess the risk for interspecies transmission in nature.

## Methods

### Viruses and cell lines

The canine isolate A/canine/Illinois/12191/2015(H3N2) (IL/15; GenBank Accession Numbers KT002533‐KT002540) was obtained from the USDA National Veterinary Service Laboratories (NVSL) in conjunction with the USDA National Animal Health Laboratory Network (NAHLN). IL/15 was originally isolated from a nasal swab from a golden retriever and passaged once in embryonated chicken eggs. The isolate from eggs was passaged in Madin‐Darby canine kidney (MDCK) cells and stored as a reagent for distribution by NVSL. The virus was passaged once more in MDCK to prepare inoculum for the study.

### Animal study

Twenty approximately 4‐week‐old crossbred healthy pigs were obtained for the challenge study. Pigs were seronegative to influenza A virus (IAV) antibodies against the nucleoprotein (NP) as measured by ELISA (Swine Influenza Virus antibody test, IDEXX, Westbrook, ME) and were negative (<10 reciprocal titer) by hemagglutination inhibition (HI) assay against IL/15 prior to challenge. Pigs were divided into three groups: non‐challenged (*n* = 5), challenged with IL/15 (*n* = 10), and contact pigs (*n* = 5). Pigs challenged with IL/15 were inoculated simultaneously via intranasal (1 ml) and intratracheal (2 ml) routes with 10^5^ 50% tissue culture infective dose (TCID_50_) per ml of virus. On 2 dpi, five naïve contact pigs were placed in a separate raised deck in the same room but with separate food and water and approximately two feet away from the inoculated group to evaluate indirect contact transmission.

Nasal swab (NS) samples were collected daily 1–5 days post‐infection (dpi) for negative control and challenged pigs, and 1–5, 7, and 9 days post‐contact (dpc) for contact pigs. Five challenged pigs were humanely euthanized and necropsied at 5 dpi, at which time bronchoalveolar lavage fluid (BALF) and tissue samples from the distal trachea and right cardiac or affected lung lobe were collected. The remaining 5/10 challenged pigs and the five contact pigs were euthanized at 14 dpi and 12 dpc, respectively, to assess seroconversion.

### Pathological examination of lungs

Tissue samples collected at necropsy were evaluated for the percentage of the lung affected by the purple‐red consolidation typical of IAV infection. The percentage of the surface of the entire lung affected by pneumonia was calculated on the basis of the weighted proportions of each lobe to the total lung volume. Microscopic lesions were evaluated and scored by a veterinary pathologist blinded to the treatment groups. The presence of IAV‐specific antigen was examined in the trachea and lung tissues using immunohistochemistry (IHC) with a mouse monoclonal antibody (clone HB65) to detect NP.

### Virus isolation and titers in NS and lung samples

For virus isolation, NS samples were filtered (0·45 μm) and plated onto confluent MDCK cells in 24‐well plates. Virus titration was performed in triplicate on confluent MDCK cells in 96‐well plates. Virus isolation and viral titer assays were performed with 1 μg/ml tosylsulfonyl phenylalanyl chloromethyl ketone (TPCK) trypsin. After 48 hours, plates were fixed with 4% phosphate‐buffered formalin and stained using a mouse monoclonal antibody (clone HB65) to detect NP.

### Serology

Collected sera were treated with receptor‐destroying enzyme (Denka Seiken, Japan), heat inactivated at 56°C for 30 minute, and adsorbed with 50% turkey red blood cells (RBCs) to remove non‐specific hemagglutinin inhibitors and natural serum agglutinins. Hemagglutination inhibition assays were performed with IL/15 as the antigen and 0·5% turkey RBCs using standard techniques.

### Nucleic acid extraction and real‐time RT‐PCR

RNA was extracted from NS and BALF samples with the MagMAX Viral RNA Isolation kit (ThermoFisher Scientific Inc, Waltham, MA). RT‐PCR was performed with the VetMAX‐Gold SIV Detection Kit following the manufacturer's instructions (ThermoFisher Scientific Inc).

## Results

This H3N2 is genetically distinct from the equine‐origin H3N8 CIV that previously circulated in the United States, but closely related to the Asian H3N2 CIV (Figure S1). The pairwise nucleotide identity between IL/15 and A/canine/Korea/01/2007(H3N2), an Asian H3N2 CIV tested experimentally in pigs,^12^ is 97·9% (PB2), 98·2% (PB1), 98·2% (PA), 98·5% (HA), 98·5% (NP), 97·4% (NA), 98·2% (MP), and 97·3% (NS). Pigs directly inoculated with IL/15 did not show significant macroscopic or microscopic lesions when compared to non‐infected pigs, and IAV antigens were not detected in lung or trachea tissues by immunohistochemistry at 5 dpi (data not shown). Virological and serological results from NS, BALF, and sera are summarized in Table 1. All NS samples were negative for IAV by virus isolation, but 2/10 samples were positive by RT‐PCR at 4 dpi. 4/5 BALF samples were positive for virus isolation, and the group mean titer was 1·39 × 10^4^ TCID_50_/ml. Serological analysis showed that 4/5 pigs seroconverted as measured with an NP ELISA and by HI against IL/15. In contrast, there was no evidence of virus replication or seroconversion in the indirect contact pigs by any sample or assay type, indicating that although direct inoculation led to limited replication in the lower respiratory tract, there was no transmission from inoculated pigs to contact pigs. Samples from non‐challenged pigs (*n* = 5) were negative for virus isolation, RT‐PCR, and HI titers.

**Table 1:** Summary of virological and serological analysis of collected samples

	Nasal Swabs		Bronchoalveolar lavage fluid		Serology	
	Virus isolation	Real‐time RT‐PCR^a^ positive	Virus isolation (TCID_50_/ml)	Real‐time RT‐PCR positive	NP ELISA positive 14 dpi/12 dpc	HI positive (≥10) 14 dpi/12 dpc
Challenged (10)	0/10	2/10 at 4 dpi	4/5 (1·39 × 10^4^ ± 1·1 × 10^4^)^b^	4/5	4/5^c^	4/5^c^
Contact (5)	0/5	0/5	–d	–d	0/5	0/5

a Samples collected at 1–5 dpi and 1–5, 7, 9 dpc were tested.

b Group mean titer and standard error of the mean. Individual titers for all five pigs were: 0, 1·00 × 10^4^, 1·78 × 10^2^, 3·16 × 10^3^, 5·62 × 10^4^ TCID_50_/ml.

c The same four pigs that were NP positive were also positive for HI titers.

d Sample not collected.

## Discussion

Closely monitoring the emergence and circulation of an IAV with antigenically distinct hemagglutinin (HA) in mammalian hosts is critical for risk assessment and outbreak preparedness. The ecology of IAV is complex, and the virus and host factors for cross‐species transmission are not fully understood. A CIV strain, previously detected in several countries in Asia, caused an outbreak in the United States in 2015. We report that the US CIV strain does not replicate efficiently in swine, especially the upper respiratory tract. Low titers of virus were detected in the lungs of 4/5 pigs. Although virus was detected by RT‐PCR in NS of 2/10 pigs, infectious virus was not isolated. Consistent with the limited replication detected in the upper respiratory tract, there was no evidence of transmission, suggesting a low risk of sustained infection in pigs.

Pigs can be transiently infected with IAV from other host lineages, and human seasonal IAV has been sporadically transmitted and sustained in swine following reassortment and adaptation. However, pigs generally have low susceptibility to direct infection with avian, equine, and canine IAV.^13^ Consistent with these findings, an *in vitro* and *in vivo* study found that pigs are more susceptible to avian and seal H3N8 than to equine and canine H3N8.^14^ An equine H3N8 was detected previously in pigs, although amino acid substitutions in the HA were observed that may be associated with overcoming host barriers,^15^ but no equine or canine IAV have become endemic in swine in recent record. Genetic markers for swine adaptation are not known; an avian‐origin H3N2 CIV from Korea [Figure S1: A/canine/Korea/01/2007(H3N2)] did not infect swine in an experimental setting,^12^ although the HA from this virus differs at nine positions in the HA1 when compared to IL/15 (Table 2). Further studies are required to identify the molecular features of IL/15 that could confer susceptibility in swine.

**Table 2:** Amino acid differences between the HA1 of A/canine/Korea/01/2007(H3N2) and A/canine/Illinois/12191/2015(H3N2)

HA1 amino acid position (H3 numbering)	A/canine/Korea/01/2007(H3N2)	A/canine/Illinois/12191/2015(H3N2)
4	P	L
25	I	M
46	S	P
128	T	A
146	G	S
218	G	E
242	V	I
261	R	H
291	D	E

This study suggests that if the novel H3N2 CIV were introduced into swine, there is low probability of sustained infection and spread. However, it is possible that CIV could acquire adaptive mutations to replicate and transmit more efficiently in swine. Transient infections could lead to reassortment between CIV and swine viruses, and a number of swine viruses possess gene segments closely related to human seasonal viruses. While CIV has been maintained in large shelters that have a high turnover rate, robust diagnostics should be continued to monitor the evolution and spread of the H3N2 CIV beyond the greater Chicago area, particularly into proximity of the large swine populations in the Midwest.

## Funding

This study was supported by USDA‐ARS, USDA‐APHIS, and by an NIH‐National Institute of Allergy and Infectious Diseases (NIAID) interagency agreement (AA14006) associated with Center of Research in Influenza Pathogenesis, an NIAID funded Center of Excellence in Influenza Research and Surveillance (HHSN272201400008C). EJA and TKA were supported in part by an appointment to the ARS‐USDA Research Participation Program administered by the Oak Ridge Institute for Science and Education through an interagency agreement between the U.S. Department of Energy and USDA under contract number DE‐AC05‐06OR23100. Mention of trade names or commercial products in this article is solely for the purpose of providing specific information and does not imply recommendation or endorsement by the U.S. Government. USDA is an equal opportunity provider and employer.

## Supporting information

Appendix S1. Methods.
Figure S1. Maximum likelihood phylogeny of 117 canine influenza H3 hemagglutinin (HA) sequences.
