# Supplementary material for: The avian‐origin H3N2 canine influenza virus that recently emerged in the United States has limited replication in swine
Source: Influenza Other Respir Viruses. 2016 May 18;10(5):429–32. doi: 10.1111/irv.12395 (PMC4947940; doi:10.1111/irv.12395)
Supplement: Supplementary file 1 — Appendix S1. Methods. Figure S1. Maximum likelihood phylogeny of 117 canine influenza H3 hemagglutinin (HA) sequences. [file IRV-10-429-s001.docx]

Supporting information

Abente et al., “The avian-origin H3N2 canine influenza virus that recently emerged in the United States has limited replication in swine.”

The supporting information contains a phylogenetic tree of 117 canine H3 HA nucleotide sequences. The methodology employed to perform the analysis is described.

Methods

Canine H3 hemagglutinin sequences were downloaded from the Influenza Research Database (http://www.fludb.org) on March 29, 2016. Alignments were generated using default settings in MAFFT v7.221 [1, 2] with subsequent manual correction. We used IQ-TREE v1.3.12 [3] to: select the best-fit model of evolution (a TPM2u model with gamma-distributed rate heterogeneity); and subsequently infer the best-known maximum-likelihood (ML) tree. Statistical support for individual branches on the phylogeny was estimated using ultrafast bootstrap approximation [4] and SH-like approximate likelihood ratio tests (SH-aLRT) [5].

**Supplementary Figure 1.** Maximum likelihood phylogeny of 117 canine influenza H3 hemagglutinin (HA) sequences. Analyses were performed employing a TPM2u model of nucleotide substitution with Γ-distributed rate variation among sites. The monophyletic clade in gray are avian-origin A/H3N2 whereas those in blue are equine-origin A/H3N8: the isolate in red and marked by a solid red square is sourced from the 2015 H3N2 canine influenza outbreak in Chicago, Illinois. Single branch SH-aLRT (first value) and bootstrap (second value) support for key nodes are shown, the scale bar represents nucleotide substitutions, and the tree is midpoint rooted for clarity.

[1] Katoh K, Misawa K, Kuma K, Miyata T. MAFFT: a novel method for rapid multiple sequence alignment based on fast Fourier transform. Nucleic acids research. 2002;30:3059-66.

[2] Katoh K, Standley DM. MAFFT multiple sequence alignment software version 7: improvements in performance and usability. Mol Biol Evol. 2013;30:772-80.

[3] Nguyen LT, Schmidt HA, von Haeseler A, Minh BQ. IQ-TREE: a fast and effective stochastic algorithm for estimating maximum-likelihood phylogenies. Mol Biol Evol. 2015;32:268-74.

[4] Minh BQ, Nguyen MA, von Haeseler A. Ultrafast approximation for phylogenetic bootstrap. Mol Biol Evol. 2013;30:1188-95.

[5] Guindon S, Dufayard JF, Lefort V, Anisimova M, Hordijk W, Gascuel O. New algorithms and methods to estimate maximum-likelihood phylogenies: assessing the performance of PhyML 3.0. Syst Biol. 2010;59:307-21.
