# Supplementary figures and images for: The avian‐origin H3N2 canine influenza virus that recently emerged in the United States has limited replication in swine
Source: Influenza Other Respir Viruses. 2016 May 18;10(5):429–32. doi: 10.1111/irv.12395 (PMC4947940; doi:10.1111/irv.12395)

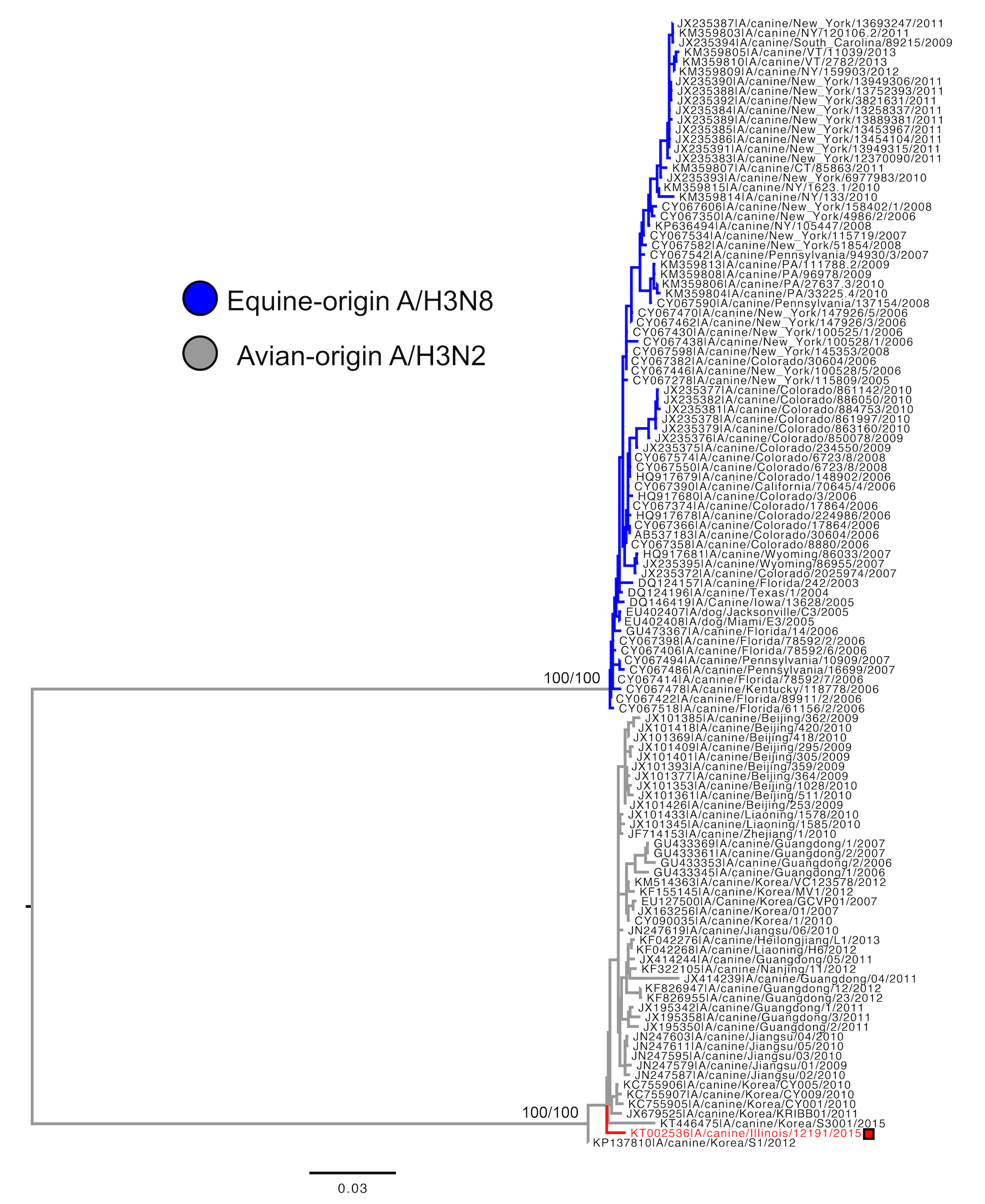

Supplement: Supplementary file 2 [file IRV-10-429-s002.tif]
